# Supplementary material for: N-glycosylation of the human β1,4-galactosyltransferase 4 is crucial for its activity and Golgi localization
Source: Glycoconj J. 2020 Aug 22;37(5):577–88. doi: 10.1007/s10719-020-09941-z (PMC7501111; doi:10.1007/s10719-020-09941-z)
Supplement: Supplementary file 1 — (DOCX 154 kb) [file 10719_2020_9941_MOESM1_ESM.docx]

**N-glycosylation of the human β1,4-galactosyltransferase 4 is crucial for its activity and Golgi localization**

Auhen Shauchuk*, Bożena Szulc*, Dorota Maszczak-Seneczko, Wojciech Wiertelak, Edyta Skurska, Mariusz Olczak

Faculty of Biotechnology, University of Wroclaw, 14A F. Joliot-Curie St., 50-383 Wroclaw, Poland

*equal contribution

Corresponding author: Mariusz Olczak Faculty of Biotechnology, University of Wroclaw, 14A F. Joliot-Curie St., 50-383 Wroclaw, Poland, e-mail: mariusz.olczak@uwr.edu.pl

**Keywords:** B4GalT4, mutagenesis, N-glycans, keratan sulfate, Golgi apparatus, CRISPR-Cas9

**Abstract**

β1,4-galactosyltransferase 4 (B4GalT4) is one of seven B4GalTs that belong to CAZy glycosyltransferase family 7 and transfer galactose to growing sugar moieties of proteins, glycolipids, glycosaminoglycans as well as single sugar for lactose synthesis. Herein, we identify two asparagine-linked glycosylation sites in B4GalT4. We found that mutation of one site (Asn220) had greater impact on enzymatic activity while another (Asn335) on Golgi localization and presence of N-glycans at both sites is required for production of stable and enzymatically active protein and its secretion. Additionally, we confirm B4GalT4 involvement in synthesis of keratan sulfate (KS) by generating A375 B4GalT4 knock-out cell lines that show drastic decrease in the amount of KS proteoglycans and no significant structural changes in N- and O-glycans. We show that KS decrease in A375 cells deficient in B4GalT4 activity can be rescued by overproduction of either partially or fully glycosylated B4GalT4 but not with N-glycan-depleted B4GalT4 version.

**Declarations:**

**Funding:**

This work was supported by the National Science Centre (NCN grant no. 2016/23/D/NZ3/01314), Krakow, Poland.

**Competing interests**

The authors declare that no competing interests exist.

**Data availability statement**

All relevant data are within the paper and its Supporting Information files.

**Ethical approval:**

This article does not contain any studies with human participants or animals performed by any of the authors.

**Author contributions**

MO, AS, BS and DMS conceived and developed the study. AS, BS and MO performed the majority of experiments and data analyses. WW performed some experiments and data analyses. ES performed some experiments. MO, AS, BS and DMS interpreted data. AS, BS and DMS drafted the manuscript. MO revised and edited the manuscript. All authors approved the final version of the manuscript.

**Introduction**

Human β1,4-galactosyltransferase 4 (B4GalT4) and other Golgi-resident B4GalT’s belong to glycosyltransferase family 7 according to classification of Carbohydrate-Active enzymes (CAZy). This family of transferases shares the topology of catalytic domain termed GT-A [1]. Seven members of B4GalTs are type II integral proteins with short N-terminal tail residing in cytosol, membrane anchor and C-terminal catalytic part of the protein constituting larger portion of the enzyme. Those galactosyltransferases mainly attach galactose (Gal) to the terminal *N*-acetylglucosamine (GlcNAc) present on nascent sugar structures of N-/O-glycans, proteoglycans and glycolipids via β1,4 linkage [2–4] what is accompanied by another common to the CAZy glycosyltransferase family 7 trait – inversion of anomeric configuration of carbon in the substrate (UDP-Gal) during catalysis. Additionally, under specific conditions, some B4GalTs can perform synthesis of lactose, which requires attachment of galactose to glucose, making the latter a second acceptor for B4GalTs [5]. Finally, initiation of proteoglycan synthesis requires galactose addition to another potential acceptor, i.e. xylose. To perform all of the above-mentioned reactions, B4GalTs utilize the activated form of galactose (UDP-Gal). For the reaction to take place, nucleotide sugar must be transported into the lumen of the Golgi apparatus (GA). Therefore, localization of B4GalT1 on the plasma membrane and its importance in the sperm-egg recognition in mice as well as evidence for secretion of B4GalT1 and B4GalT4 raises a question about additional functions of those enzymes [5–9]. The functions of B4GalTs residing outside of the Golgi apparatus are yet to be clarified, although the importance of secreted B4GalTs might lie outside of glycosylation, as was observed for the secreted version of GlcNAc transferase Mgat5 [11].

Previously, it was postulated that the first cloned B4GalT, i.e. B4GalT1, was the only transferase responsible for β1,4-galactosylation, being β1,4-galactosyltransferase unique for glycoconjugates [12, 13]. However, studies on β1,4-galactosylated structures as well as levels of β1,4-galactosyltransferase activity in different tissues led researchers into cloning of several other B4GalTs with intertwining activities. Recent studies presenting mutant cells lacking different combinations of B4GalT1-4 clearly re-establish B4GalT1 as the major galactosyltransferase for synthesis of N-glycans [14]. Nevertheless, activities of B4GalTs overlap significantly, which impedes the assignment of different enzymes with specific non-redundant biological roles. Multiple nucleotide sequence alignment of B4GalTs shows little similarity although such amino acid comparison shows higher consensus in the central region [4].

Several works put specific emphasis on the importance of B4GalT4. McDonald et al. presented the influence of its 40% knock-down in Chinese hamster ovary (CHO) cells on the increase of the N-glycan branching [15]. However, this effect was not reproduced in cells with knock-out combinations of B4GalT4 with B4GalT1-3 [14]. Furthermore, a strong link between overexpression of B4GalT4 in colorectal cancer cells and metastatic potential was demonstrated and another study on tumor tissues of patients with endometrial cancer found a significant correlation of B4GalT4 and B4GalT1 transcript levels with likelihood of successful treatment [16, 17]. A recent study suggested a genomic explanation for such up-regulation of B4GalT4 in colorectal cancer cells [18].

*In vitro* enzymatic activities of B4GalTs were the subject of several studies. In those works B4GalT4 was clearly differentiated from other B4GalTs by the lack of activity towards asialo, agalacto-transferrin [1, 3]. Additionally, B4GalT4 was shown not to be inhibited by high concentrations of GlcNAc, although its kinetic properties towards the latter indicate much lower activity compared to other B4GalTs [5]. It was also demonstrated *in vitro* that B4GalT4 acts on the sulfated acceptors with much higher potency than other family members. That fact suggests the involvement of B4GalT4 in biosynthesis of keratan sulfate (KS) and 6-*O*-sulfated *N*-acetyllactosamine moiety [19]. Additionally, in an *in vitro* study, B4GalT4 together with transferase of GlcNAc and sulfotransferase were found to synthesize KS and silencing of the B4GalT4 transcript in MDCK cell cultures caused depletion of KS proteoglycans [20, 21]. Finally, *in vitro* studies of B4GalT4 activity suggested its involvement in synthesis of O-linked poly-*N*-acetyllactosamine chains [22].

Analysis of genomic localization shows that different B4GalTs are probably derived by gene duplication with consequent alteration in the primary sequence of individual members. Besides a short N-terminal transmembrane helix all B4GalTs possess 4 conserved cysteine residues and several homologous sequence motifs (DVD, FGGVS and WGWGGEDDD). B4GalT2-6 also share one N-glycosylation site near the C-terminus of the protein [13]. Based on the mutagenesis studies of B4GalT1 it is possible to assume that C-terminal part of the transferase bearing mentioned N-glycosylation site is the region responsible for the substrate recognition [23].

All galactosyltransferases utilize UDP-galactose (UDP-Gal) as a substrate. This nucleotide sugar is delivered into the ER/Golgi lumen by a specific member of the solute carrier 35 (SLC35) family, namely SLC35A2 (UDP-Gal transporter, UGT) [24]. Two splice variants of this protein have been identified: UGT1, which resides in the Golgi complex, and UGT2, which localizes both to the ER and Golgi membranes [25]. We recently demonstrated that UGT1 interacts with B4GalT1, which raises the question of whether other B4GalTs also display such ability [26].

In the present study, with the help of mutagenesis, immunofluorescence and CRISPR-Cas9 technology we demonstrate that B4GalT4 possesses two N-glycans at Asn220 and Asn335. Removal of either of the two sites abolished enzymatic activity of B4GalT4 observed *in vitro* but had different effects on the subcellular localization and ability to restore the wild-type phenotype in A375 cell cultures depleted in endogenous *B4GALT4*. Hence, we postulate non-equivalent roles of these two N-glycans in modulation of B4GalT4 function. Moreover, we show that B4GalT4 interacts with SLC35A2 and this phenomenon is also affected by the loss of N-glycans from the former.

**Materials and methods**

*Cell cultures and stable transfection*

A375 and HEK293T cells obtained from the American Type Culture Collection were cultured in Dulbecco’s Minimum Eagle Medium (DMEM High Glucose, Biowest) under 5% CO_2_ and 37 °C. The medium was supplemented with 10% fetal bovine serum (FBS), 100 U/ml penicillin, 100 μg/ml streptomycin and 4 mM L-glutamine. Stable transfections with pSelect plasmids for protein expression were performed using the FuGENE HD transfection reagent (Promega) according to the manufacturer’s protocol. Selection of stable transfectants overexpressing different B4GalT4 variants was done in the complete media supplemented by 400 μg/ml of zeocin (InvivoGen). Analysis of stable transfectants was done by Western blotting and indirect fluorescent staining. After the selection the cell culture was maintained with a two-fold reduced zeocin concentration.

*Cloning and mutagenesis*

cDNA encoding human B4GalT4 protein (NCBI accession number NM_212543) was synthesized from the total RNA isolated from HeLa cells. All constructs were cloned using restriction enzymes except for pSelect-blasti. For cloning with restriction enzymes, PCR was performed with the cDNA using primers listed in Table S1. Products were then purified and digested with BamHI and NheI enzymes and finally cloned into the pSelect-zeo vector (InvivoGen). The QuikChange Multi Site-Directed Mutagenesis Kit (Agilent Technologies) was used to introduce the following mutations into the cloned wild type B4GalT4: T6A (A16G), T222A (A664G), T337A (A1009G) and T222A/T337A. Resulting constructs were named accordingly as MT1-3 and MT23. Verification by DNA sequencing was performed for each construct (Microsynth). All versions of B4GalT4 were than consequently cloned into the pSelect-NHA from pSelect-zeo with the RF-cloning strategy [27]. Schematic representation of constructs is available in Supplementary Data (Fig. S2a).

*Construction and analysis of B4GalT4 knock-out cell line*

Generation of B4GalT4-deficient cell lines was performed with two strategies alongside. Both of strategies relied on transfection with combination of plasmids. Ready to use Santa Cruz Biotechnology CRISPR kit used just one antibiotic resistance for selection of transfected cells, while Addgene plasmids were designed to use combination of antibiotic resistance. According to the manufacturer's instructions (Santa Cruz Biotechnology) A375 and HEK293T cells were transfected with a supplied mixture of human B4GalT4 double nickase plasmids (sc-407251-NIC). Next, selection was performed with 0.5 µg/ml of puromycin (InvivoGen). The second strategy involved cloning of gRNA into modified plasmids px462 and px459 (Addgene). Design of gRNA was performed with the help of the online tool Atum [www.atum.bio/]. Primer sequences corresponding to the cloned gRNA can be found in Table S1. Selection of cells was performed with a combination of puromycin (1 µg/ml) and zeocin (400 µg/ml). Isolated clones were screened for presence of the *B4GALT4* transcript by one-step RT-PCR with HEK293T and/or A375 wild type cells as the controls. Primer sequences are listed in Table S1. Consequently, to verify mutations of selected cells, mixtures of RT-PCR products were cloned into the pJET1.2/blunt vector (Thermo Fisher Scientific) for DNA sequencing (Microsynth).

*Immunofluorescent imaging*

Poly-L-lysine (0.01%) coated glasses were used for culturing cells prior to staining. Cells were fixed on the second day of culture with 4% paraformaldehyde (PFA) in PBS (phosphate-buffered saline) added to the medium for 2 minutes and for the following 10 minutes with PFA applied after discarding the previous mixture from cells. Afterwards cells were washed 3 times for 5 minutes with PBS. To block non-specific binding sites 1 h incubation with 1% (w/v) BSA and 0.1% (w/v) saponin in PBS was performed. After blocking, cells were incubated for 1 h with primary antibodies (Table S3) at 37°C. Washing (3 x 5 min) was performed with blocking solution. Subsequently, slides were incubated with secondary antibodies (Table S3) for 1 h at 37°C. Counterstain with 4ʹ,6-diamidino-2-phenylindole (DAPI, Sigma-Aldrich) was performed for nuclei visualization. For staining finalization, fluorescence mounting medium (Dako) was used to mount slides onto glass coverslips. Slides were examined with a ZEISS LSM 510 confocal microscope and pictures were analyzed using ImageJ software (NIH).

*Western blotting, secretion analysis and proteasome inhibition*

For lysate preparations, cells were washed with PBS, harvested, centrifuged for 5 minutes at 200 g at RT, washed with PBS and centrifuged once again. Cell pellets were lysed for 15 minutes at RT in lysis buffer [CelLytic M reagent (Sigma-Aldrich), 1 mM EDTA, proteinase inhibitors (Bimake)]. Lysates were then centrifuged for 15 minutes at 14 000 g at RT and pellets were discarded. Protein concentration was determined using Roti-Quant reagent (Roth) and samples were equilibrated with lysis buffer. After protein equilibration, dedicated samples were incubated with 2500 U of peptide:N-glycosidase F (PNGase F) in lysis buffer at 37°C overnight. For SDS-PAGE lysates were mixed with sample buffer (1.5 M Tris-HCl, 10% glycerol, 45 mM SDS, 5 mg/ml DTT, bromophenol blue, pH 6.8) and denatured for 10 minutes at 95°C. Proteins were resolved in acrylamide gels and transferred onto nitrocellulose membrane (GE Healthcare). Blocking was performed for 5 h at RT in 5% powdered fat-free milk in PBS with 0.1% Tween-20 (Roth). Subsequently, membranes were incubated overnight at 4°C with primary antibodies (Table S3) in blocking solution. On the following day, membranes were rinsed with 1% powdered fat-free milk in PBS with 0.1% Tween-20 and incubated for 1 h at RT with secondary antibodies coupled with horseradish peroxidase (Table S3). Visualization of immunoreactive proteins was carried out with a chemiluminescence system (PerkinElmer, Waltham, MA, USA).

For proteasome inhibition, cells were incubated for 6 h with complete DMEM medium supplemented with 8 µM MG132 (M7449 Sigma-Aldrich).

Secretion analysis was performed by culturing HEK293T cells overexpressing different B4GalT4 versions in Opti-MEM without FBS for 24 h in standard conditions. Conditioned media were collected and concentrated by Amicon Pro 10 kDa cut-off membranes. Analysis of concentrates was done as described above by Western blotting with anti-B4GalT4 and anti-Mgat5 antibodies for protein assessment and the loading control, respectively.

*Enzymatic assay*

Recombinant B4GalT4 (Novoprotein) was used for establishing *in vitro* conditions for analysis of enzymatic activity. M592 pentasaccharide (V-labs Fig. S2b) was labeled with 2-aminobenzamide (2-AB) and used as an acceptor. Reaction was performed with 130 ng of acceptor in 50 mM Tris-HCl, 5 mM MnCl_2_, 5mM CaCl_2_, 0.5% Tween-20, pH 7.6, overnight at 37°C. Reaction products were analyzed by RP-HPLC. Time of separation on GlycoSepN column = 110 minutes; time range was set from 2000 to 10000 (approximately from 17.7 min to 73.3 min). For lysate-derived samples, Y axes were normalized due to background line flow in order to have comparable signals. Total range is preserved in all samples, except the recombined enzyme, where the signal was much higher.

*Immunoprecipitation*

Cells from two 100mm culture plates were harvested and suspended in immunoprecipitation buffer (50 mM Tris-HCl, 150 mM NaCl, 0.5% Tween-20 with proteinase inhibitors) and mildly sonicated. Ani-HA magnetic beads (Bimake) were used according to the manufacturer’s protocol. For Western blotting detection a part of the beads was mixed with SDS-PAGE sample buffer and incubated for 10 minutes at 95°C. For *in vitro* enzymatic assay enzymes immobilized on beads were used.

*Analysis of glycoconjugates*

For N-glycan analysis, proteins from cell lysates were precipitated by overnight incubation with acetone at -20°C. Precipitates were then solubilized and overnight digestion with PNGase F at 37°C was performed. Released N-glycans were purified with Supelclean LC18 (Supelco) columns. Obtained samples were vacuum-dried overnight and subsequently labeled with 2-AB for 3 h at 65°C. Final purification of labeled glycans prior to HPLC analysis was performed using cellulose chromatography. Labeled sugars were applied onto the column in a high concentration of acetonitrile and eluted with water. O-glycans were analyzed by an adopted method described by Kudelka et al. [28]. Analysis of proteoglycans was performed by Western blotting as described above with antibody specific for keratan sulfate (Table S3).

Isolated glycans were analyzed by MALDI-TOF at the Center for Molecular and Macromolecular Research, Polish Academy of Sciences, Lodz, Poland. 2.5-dihydroxybenzoic acid (Sigma) in 20 mM sodium acetate in 20% MeCN was used as a matrix. Collected results were plotted in Microsoft Office and graphics were created in Affinity Designer (Serif Europe).

*Split luciferase complementation assay*

B4GalT4-deficient HEK293T cells (2 × 10^4^) were seeded in a complete growth medium onto a 96-well plate with white polystyrene wells and a flat transparent bottom (Greiner Bio-One, Kremsmünster, Austria). 20–24 h after plating the cells were transfected with appropriate combinations of plasmids (25 ng/well of each plasmid, 50 ng/well in total, Table S2) using the FuGENE HD transfection reagent. 2-3 h before the measurement the conditioned medium was replaced with the serum-free OPTI-MEM medium (Life Technologies, CA, USA). Immediately before the measurement, Live Cell Reagent (Promega) was added to all wells, according to the manufacturer's instructions. Cell-derived luminescence was recorded using a Glomax Discover Microplate Reader (Promega). Each tested combination was accompanied by the respective negative control comprising SLC35A2 fused with the large NanoLuc subunit combined with the HaloTag (a recombinant protein not synthesized by mammalian cells, thus not interacting with proteins of mammalian origin) fused with the small NanoLuc subunit (Promega). For statistical analysis of data one-way ANOVA with the Tukey post-hoc test was used. All analyses were performed with GraphPad Prism (GraphPad Software, CA, USA). Statistical significance was assigned to p-value <0.05. Only results exceeding the respective negative controls by at least 10-fold were considered indicative of an interaction, as suggested by the manufacturer.

**Results**

*Investigation of B4GalT4 glycosylation sites*

Prediction of glycosylation sites for B4GalTs was based on the amino acid sequence. Since B4GalTs are thought to be type II transmembrane proteins, some of the foreseen sites would be on the cytosolic side of the Golgi membrane. B4GalT4 has 3 potential glycosylation sites (Asn4, Asn220 and Asn335). However, Asn4 resides upstream of the transmembrane domain. Thus, in the case of correct prediction of protein topology only 2 of 3 sites could be occupied by glycans [13]. For examination of glycosylation sites, we mutated all the glycosylation consensus motifs (either individually or in combinations) by threonine to alanine substitutions. For preliminary assessment, we transfected HEK293T cells with plasmids containing wild-type protein (myc-B4GalT4) or different mutants (myc-MT1–3). Obtained lysates from cells overexpressing B4GalT4 variants were analyzed using Western blotting with anti-*c*-myc and anti-HA antibodies (Fig. 1a). We treated lysates with peptide:N-glycosidase F (PNGase F) to clarify the nature of the apparent mass shift. The relative decrease of molecular weight of bands corresponding to B4GalT4 mutated variants MT2 and MT3 as well as samples treated with PNGase F indicates that B4GalT4 is indeed glycosylated. Some unglycosylated forms present in cells overexpressing all B4GalT4 variants are most likely to be the result of high levels of protein expression and transient transfection. High levels of protein overexpression might be overloading the glycosylating machinery, leaving some portion of the produced protein unglycosylated [29]. Additionally, we performed next steps of characterization of B4GalT4 glycosylation variants in A375 B4GalT4 KO cells that are described later in text. We again transfected cells with plasmids bearing individual mutations. Mass shifts of bands corresponding to cells overexpressing MT2 and MT3 but not MT1 clearly indicate that in fact only Asn220 and Asn335 are glycosylated (Fig.1b). Absence of glycan on Asn4 is expected from cytosolic localization of the B4GalT4 N-terminus.

*Impact of glycan removal on subcellular localization of B4GalT4*

B4GalTs are assumed to localize in the Golgi membrane. Therefore, we performed staining of cells overproducing wild type (WT) or mutant versions of B4GalT4 with Golgi and ER markers (Fig. 2, Fig. S1). As expected, non-mutated protein localized to the Golgi apparatus. However, both the distribution and the protein level observed in Western blotting of non-glycosylated B4GalT4 (MT23) were severely impaired compared to those of the wild type. The signal corresponding to MT23 was no longer localized in the Golgi membrane but instead was scattered throughout the cell. While depletion of the Asn220 glycosylation site alone seemed to have little or no effect on protein localization, removal of the Asn335 glycosylation site caused a similar effect in subcellular localization to MT23 although in less pronounced way. Distribution of glycosylation mutants overlaid with the ER marker although not fully (Fig. S1). No overlay with early endosome and lysosome markers was observed (data not shown). Therefore, we hypothesized that the non-glycosylated B4GalT4 variants could be partially exported to the cytosol for proteasome-mediated degradation, which seemed more likely since treatment of cells with the proteasomal inhibitor MG-132 for 6 h before staining caused reversion of the signal derived from mutant variants from the dispersed pattern towards the more concentrated, perinuclear one (Fig. S6).

*Secretion of B4GalT4 and its unglycosylated variants*

Prompted by literature [6, 25] and our preliminary Western blotting analysis we investigated B4GalT4 secretion. Indeed, non-mutated B4GalT4 was shown to be secreted to the medium by Western blotting detection with two antibodies (Fig. 3). In contrast, none of the mutated versions was found in the medium. It is even more interesting that although the MT2 unglycosylated variant is found at similar subcellular locations as non-altered B4GalT4. Thus, we propose that N-glycans on both consensus motifs are critically important for B4GalT4 secretion.

*In vitro activity of B4GalT4 and its unglycosylated variants*

During our preliminary analysis we observed B4GalT4 activity towards labeled sugar structure similar to nascent N-glycan with two terminal GlcNAc residues. Hence, we established conditions for enzymatic assay *in vitro* using the recombinant B4GalT4 (Materials and Methods). To validate the impact of glycan removal on B4GalT4 activity we performed immunoprecipitation and purified proteins were subjected to analysis (Fig. 4). While activity of non-mutated protein was apparent, the results obtained for MT2 and MT3 variants were comparable with those of the negative control, although we were unable to purify considerable amounts of MT23 for analysis. Therefore, we conclude that the glycan part of B4GalT4 might be crucial for its enzymatic activity.

*In vivo activity of B4GalT4 and its unglycosylated variants*

To investigate the importance of glycans for enzymatic activity towards endogenous acceptors we generated HEK293T and A375 cells depleted in functional B4GalT4 using the CRISPR-Cas9 approach. Obtained knock-out cell lines (KO) were verified using sequencing of transcripts (Fig. S2). Interestingly, we observed no significant differences between N- and O-glycans of wild type and HEK293T and A375KO cells (Fig. S3 and S4). Since two major classes of glycoconjugates did not show alterations in KO cells, we additionally analyzed keratan sulfate in A375 cells (Fig. 5). HEK293T cells were not used in the latter analysis because of the low keratan sulfate content.

Strikingly, in the case of A375 cells depletion of the *B4GalT4* gene caused a drastic decrease in keratan sulfate proteoglycans. Correction of the observed A375 KO phenotype could be done with overproduction of unchanged B4GalT4. Overproduction of mutated B4GalT4 versions caused a similar correction of KO phenotype but only in the case of the MT3 version. Overproduction of MT2 in KO cells did show an increase of keratan sulfate proteoglycans in cells but not to levels similar to the ones caused by either B4GalT4 or MT3. B4GalT4 depleted in both N-glycans (MT23) was not able to show a similar correction ability in A375 KO cells. Additionally, we performed immunofluorescence staining in A375 B4GalT4 KO cells overproducing different versions of B4GalT4 with the markers of the Golgi and ER showing previously observed localizations as in HEK293T cells (Fig. S5).

*Split luciferase complementation assessment of B4GalT4 interaction with SLC35A2*

We applied a recently developed, commercially available procedure based on the split luciferase complementation assay to assess interactions of different B4GalT4 versions overproduced in HEK293T cells with other proteins. Since another member of the B4GalT family, i.e. B4GalT1, was recently reported to interact with the UDP-Gal transporter (UGT) [26], we examined the ability of the latter to associate with B4GalT4 and its mutant variants using the split luciferase complementation assay mentioned above. As the wild type B4GalT4 displayed Golgi localization, we employed the first splice variant of the UDP-Gal transporter (UGT1) that resides exclusively in the Golgi apparatus [25]. Having demonstrated an association of the wild type B4GalT4 with UGT1 we assumed that the B4GalT4 variants unable to reach the target destination would not make any physical contacts with the transporter. Indeed, MT2, which is the only variant that retains Golgi localization, could interact with UGT1 to a greater extent than the wild type enzyme (Fig. 6). In contrast, MT3 and MT23, both of which delocalize to a significant extent, were unable to associate with UGT1. Therefore, the results of the split luciferase complementation assay are in agreement with the localization data.

**Discussion**

N-glycans are known to modulate protein levels, subcellular localization, conformation and function, although the specific impact is often unique to the modified protein. Herein we identified two occupied N-glycosylation sites on the 4^th^ member of the β1-4 galactosyltransferase family at Asn220 (MT2) and Asn335 (MT3). In an attempt to identify the roles that N-glycans at the found locations may play in mature protein we overproduced mutated versions of B4GalT4 lacking either individual or both N-glycans in HEK293T and A375 cell lines. *In vitro* analysis demonstrated no activity of immunopurified B4GalT4 single mutants. Although we purified enzymes in native conditions and hence final products used in the analysis could be protein complexes rather than individual enzymes, commercially available purified recombinant B4GalT4 from the HEK293T cell line was able to produce sugar structures of higher mass, which allowed us to assume that galactosylation performed in the *in vitro* assay requires only B4GalT4. To the best of our knowledge this is the first time that the N-glycans of B4GalT4 have been shown to contribute to production of a stable, enzymatically active protein [31].

In this study we showed the dependence of proper B4GalT4 localization in the Golgi complex and its secretion from the cells on the presence of N-glycans, upon removal of which high levels of B4GalT4 show delocalization to the ER and presumably cytosol for proteasomal degradation. Interestingly, in contrast to subcellular localization, secretion was not only diminished but completely abolished in the case of depletion of B4GalT4 of any N-glycan, leading us to assume that although ER to Golgi trafficking is facilitated by at least one N-glycan attached at Asn335, its secretion strictly requires the presence of both N-glycans.

Recently, the importance of homo- or heteromeric complexes in glycosylation has gained more attention [32]. To investigate any effect possibly involving such complexes by removal of glycans on the surface of B4GalT4, we generated and characterized HEK293T and A375 cell lines depleted in functional endogenous *B4GALT4*. We observed no significant change in N-glycosylation of KO cell lines, confirming the results of the latest research on the Chinese hamster ovary cell line depleted in combinations of B4GalT1-4 [14, 33]. Surprisingly, we were not able to note any differences in O-glycans although in this regard the role of B4GalT4 could be expected to be more pronounced [4, 22]. In *in vitro* studies B4GalT4 showed a preference towards sulfated acceptors as well as being found to be capable of *in vitro* synthesis of short keratan sulfate chains; therefore we investigated the impact of *B4GALT4* removal in cell cultures on keratan sulfate production [19, 20]. Strikingly, KO cell cultures showed dramatic reduction of keratan sulfate proteoglycans. Based on such observation and correction of the phenotype by unchanged B4GalT4 overproduction in KO cells, we claim that B4GalT4 is essential for the synthesis of keratan sulfate in A375 cells.

Additionally, not all forms of B4GalT4 showed similar levels of correction with the mutant version depleted of both glycans presenting only neglectable levels. Overproduction of the mutant B4GalT4 version lacking a glycosylation site at Asn220 (MT2) showed significantly lower ability to correct the KO phenotype in A375 KO cell cultures. Although, to our knowledge, currently there are no available studies linking structural elements of B4GalT4 to its enzymatic activity, sequence comparison of B4GalTs shows that this glycosylation site of B4GalT4 is positioned closely to conserved regions found to be engaged in catalytic activity and acceptor recognition in B4GalT1 [13, 34, 35]. Interestingly, B4GalT4 is the only B4GalT to possess an N-glycosylation site near this regionIn parallel, the second N-glycosylation site of B4GalT4 is conserved by homology among B4GalT2-6 besides B4GalT1 and B4GalT7. Additionally, depletion of that C-terminal N-glycan produced a transferase able to correct the A375 KO phenotype, presumably leaving enzymatic activity intact while causing partial delocalization.

Since recently it was revealed that some transferases can form multi-enzyme complexes with nucleotide sugar transporters, we decided to explore B4GalT4 interactions with the Golgi-residing UDP-Gal transporter (UGT1) and how it can be affected by glycan removal on transferase [36]. Interestingly, observed interactions between transferase and transporter shown by the split luciferase assay were abolished in the case of B4GalT4 delocalized versions (MT3 and MT23) while removal of the N-glycosylation site essential for enzymatic activity (MT2) showed an increase of them. Those results combined with observed complete depletion of secretion of all B4GalT4 mutant versions led us to hypothesize that the lack of secretion of MT3 and MT23 results from their retention in the ER, while MT2, although reaching the Golgi complex, is retained in this organelle and thus prevented from secretion, as reflected by a significantly tighter association with UGT1.

Interestingly, in spite of expectations in our case we observed ability of B4GalT4 to be involved in synthesis of keratan sulfate while being depleted of interaction with UGT1. It might be that disruption of interactions with other transferases rather than transporter would be correlated with loss of restoration of wild-type phenotype in *B4GALT4-*depleted cells. Such an assumption is based on the remark by Hassinen and Kellokumpu on the importance of B4GalT1 heteromers with another glycoenzyme, ST6Gal1, for their enzymatic activity. [37]. Additionally, the complexity of keratan synthesis may require similar cooperation between transferases for UDP-GlcNAc, UDP-Gal and sulfotransferases. Identified *in vitro* study candidates for such enzymes were B4GalT4, B3GnT7, CGn6ST and KSGalT6ST [19, 20, 38]. With previous findings in mind it could be interesting to investigate whether the N-glycan moiety of B4GalT4 could take part in interactions between those enzymes.

Since depletion of the Asn220 N-glycosylation site caused significant impairment of the ability to restore keratan synthesis in A375 KO cell cultures while allowing the protein to preserve proper localization, and depletion of the Asn335 glycosylation site disturbed localization and interactions with UDP-Gal transporter, we see corresponding N-glycans as activity facilitating (Asn220) or localization facilitating (Asn335). Additional support for such a claim is conservation by homology of the Asn335 N-glycosylation site between B4GalT2-6 while conservation of activity facilitating Asn220 N-glycan is not required because of different B4GalT activity properties. Decrease of enzymatic activity after N-glycan removal is a common phenomenon described for such glycoenzymes as β1,4-*N-*acetylgalactosaminyltransferase 1, β1,3-*N-*acetylglucosaminyltransferase 2, β1,4-*N-*acetylglucosaminyltransferase 3, ⍺1,3-fucosyltransferase 4 and many others [39–42]. As reviewed and summarized by Skropeta, in most cases the explanation for enzymatic activity loss upon impairment of N-glycosylation is an assumption of destabilization of active conformation [43]. This explanation could be supported by a recent study of collection of X-ray glycosylated and deglycosylated protein structures from PDB. Lee and colleagues showed that N-glycosylation does not significantly affect global protein structure of folded proteins but rather causes a decrease in protein dynamics propagated from the N-glycosylation site throughout the protein, causing a general increase of stability [44].

Impairment of secretion of N-glycan-depleted enzymes is mostly a consistent observation for most studies focused on glycoenzymes although with rare exceptions, e.g. for ⍺2,3-sialyltransferase 5 [45]. Alongside loss of secretion a partial or complete loss of Golgi localization after deglycosylation is commonly observed [43]. Interestingly, while localization of some enzymes could be unaffected by N-glycan removal, as observed in the case of β1,4-*N*-acetylgalactosaminyltransferase 1 and ⍺1,3-fucosyltransferase 4 [39, 42]. Additionally, among other galactosyltransferases it was shown that activity and proper intercellular trafficking of β1,3-galactosyltransferase 4 depends on its N-glycosylation while β1,4-galactosyltransferase 1 devoid of its N-glycans could be produced as enzymatically active and soluble protein [46, 47]. Such studies are hard to unify since the effects on trafficking and activity might lie in change of solubility, formation of aggregates and kin interactions; therefore more detailed studies are needed to investigate the direct role of N-glycans in protein subcellular localization of proteins of interest.

Our findings demonstrate that N-glycans of B4GalT4 are important for protein activity both in *in vitro* assays and in cell cultures. We also observed modulation of B4GalT4 interaction with the SLC35A2 UDP-galactose transporter by the presence of N-glycans. Summarizing, in the present study we show for the first time the unequally distributed importance of B4GalT4 N-glycans and their modulation of the enzyme activity both *in vitro* and in cell cultures.

**References**

1. Lombard, V., Golaconda Ramulu, H., Drula, E., Coutinho, P.M., Henrissat, B.: The carbohydrate-active enzymes database (CAZy) in 2013. Nucleic Acids Res. 42, D490-495 (2014). https://doi.org/10.1093/nar/gkt1178

2. Guo, S., Sato, T., Shirane, K., Furukawa, K.: Galactosylation of N-linked oligosaccharides by human  -1,4-galactosyltransferases I, II, III, IV, V, and VI expressed in Sf-9 cells. Glycobiology. 11, 813–820 (2001). https://doi.org/10.1093/glycob/11.10.813

3. Seko, A., Dohmae, N., Takio, K., Yamashita, K.: Beta 1,4-galactosyltransferase (beta 4GalT)-IV is specific for GlcNAc 6-O-sulfate. Beta 4GalT-IV acts on keratan sulfate-related glycans and a precursor glycan of 6-sulfosialyl-Lewis X. The Journal of biological chemistry. 278, 9150–8 (2003). https://doi.org/10.1074/jbc.M211480200

4. Schwientek, T., Almeida, R., Levery, S.B., Holmes, E.H., Bennett, E., Clausen, H.: Cloning of a novel member of the UDP-galactose:beta-N-acetylglucosamine beta1,4-galactosyltransferase family, beta4Gal-T4, involved in glycosphingolipid biosynthesis. The Journal of biological chemistry. 273, 29331–40 (1998). https://doi.org/10.1074/JBC.273.45.29331

5. Sato, T., Aoki, N., Matsuda, T., Furukawa, K.: Differential effect of alpha-lactalbumin on beta-1,4-galactosyltransferase IV activities. Biochemical and biophysical research communications. 244, 637–641 (1998). https://doi.org/10.1006/bbrc.1998.8327

6. Lopez, L.C., Youakim, A., Evans, S.C., Shur, B.D.: Evidence for a molecular distinction between Golgi and cell surface forms of β1,4-galactosyltransferase. Journal of Biological Chemistry. 266, 15984–15991 (1991)

7. Gopal, S.K., Greening, D.W., Mathias, R.A., Ji, H., Rai, A., Chen, M., Zhu, H.-J., Simpson, R.J.: YBX1/YB-1 induces partial EMT and tumourigenicity through secretion of angiogenic factors into the extracellular microenvironment. Oncotarget. 6, 13718–30 (2015). https://doi.org/10.18632/oncotarget.3764

8. Witzke, K.E., Rosowski, K., Müller, C., Ahrens, M., Eisenacher, M., Megger, D.A., Knobloch, J., Koch, A., Bracht, T., Sitek, B.: Quantitative Secretome Analysis of Activated Jurkat Cells Using Click Chemistry-Based Enrichment of Secreted Glycoproteins. Journal of Proteome Research. 16, 137–146 (2017). https://doi.org/10.1021/acs.jproteome.6b00575

9. Huntley, R.P., Sawford, T., Mutowo-Meullenet, P., Shypitsyna, A., Bonilla, C., Martin, M.J., O’Donovan, C.: The GOA database: Gene Ontology annotation updates for 2015. Nucleic Acids Research. 43, D1057–D1063 (2015). https://doi.org/10.1093/nar/gku1113

10. Scully, N.F., Shaper, J.H., Shur, B.D.: Spatial and temporal expression of cell surface galactosyltransferase during mouse spermatogenesis and epididymal maturation. Developmental Biology. 124, 111–124 (1987). https://doi.org/10.1016/0012-1606(87)90464-7

11. Saito, T., Miyoshi, E., Sasai, K., Nakano, N., Eguchi, H., Honke, K., Taniguchi, N.: A secreted type of beta 1,6-N-acetylglucosaminyltransferase V (GnT-V) induces tumor angiogenesis without mediation of glycosylation: a novel function of GnT-V distinct from the original glycosyltransferase activity. J. Biol. Chem. 277, 17002–17008 (2002). https://doi.org/10.1074/jbc.M200521200

12. Shaper, N.L., Shaper, J.H., Meuth, J.L., Fox, J.L., Chang, H., Kirsch, I.R., Hollis, G.F.: Bovine galactosyltransferase: identification of a clone by direct immunological screening of a cDNA expression library. Proceedings of the National Academy of Sciences of the United States of America. 83, 1573-1577. (1986). https://doi.org/2419911

13. Amado, M., Almeida, R., Carneiro, F., Levery, S.B., Holmes, E.H., Nomoto, M., Hollingsworth, M.A., Hassan, H., Schwientek, T., Nielsen, P.A., Bennett, E.P., Clausen, H.: A Family of Human β4-Galactosyltransferases. Journal of Biological Chemistry. 273, 12770–12778 (1998). https://doi.org/10.1074/jbc.273.21.12770

14. Bydlinski, N., Maresch, D., Schmieder, V., Klanert, G., Strasser, R., Borth, N.: The contributions of individual galactosyltransferases to protein specific N-glycan processing in Chinese Hamster Ovary cells. Journal of Biotechnology. 282, 101–110 (2018). https://doi.org/10.1016/J.JBIOTEC.2018.07.015

15. McDonald, A.G., Hayes, J.M., Bezak, T., G uchowska, S.A., Cosgrave, E.F.J., Struwe, W.B., Stroop, C.J.M., Kok, H., van de Laar, T., Rudd, P.M., Tipton, K.F., Davey, G.P.: Galactosyltransferase 4 is a major control point for glycan branching in N-linked glycosylation. Journal of Cell Science. 127, 5014–5026 (2014). https://doi.org/10.1242/jcs.151878

16. Chen, W.-S., Chang, H.-Y., Li, C.-P., Liu, J.M., Huang, T.-S.: Tumor beta-1,4-galactosyltransferase IV overexpression is closely associated with colorectal cancer metastasis and poor prognosis. Clinical cancer research : an official journal of the American Association for Cancer Research. 11, 8615–22 (2005). https://doi.org/10.1158/1078-0432.CCR-05-1006

17. Wang, Z.-H., Zhang, Y.-Z., Wang, Y.-S., Ma, X.-X.: Identification of novel cell glycolysis related gene signature predicting survival in patients with endometrial cancer. Cancer Cell Int. 19, 296 (2019). https://doi.org/10.1186/s12935-019-1001-0

18. Sugiyama, A., Fukushima, N., Sato, T.: Transcriptional mechanism of the β4-galactosyltransferase 4 gene in SW480 human colon cancer cell line. Biological & Pharmaceutical Bulletin. 40, 733–737 (2017). https://doi.org/10.1248/bpb.b17-00064

19. Seko, A., Dohmae, N., Takio, K., Yamashita, K.: Beta1,4-Galactosyltransferase ( beta4GalT)-IV Is Specific for GlcNAc 6-O-Sulfate. Biochemistry. 278, 9150–9158 (2003)

20. Kitayama, K., Hayashida, Y., Nishida, K., Akama, T.O.: Enzymes responsible for synthesis of corneal keratan sulfate glycosaminoglycans. J. Biol. Chem. 282, 30085–30096 (2007). https://doi.org/10.1074/jbc.M703695200

21. Maszczak-Seneczko, D., Sosicka, P., Olczak, T., Jakimowicz, P., Majkowski, M., Olczak, M.: UDP-N-acetylglucosamine transporter (SLC35A3) regulates biosynthesis of highly branched N-glycans and keratan sulfate. Journal of Biological Chemistry. 288, 21850–21860 (2013). https://doi.org/10.1074/jbc.M113.460543

22. Ujita, M., Mcauliffe, J., Almeida, R., Hindsgaul, O., Clausen, H., Fukuda, M., Schwientek, T.: Synthesis of Poly- N -acetyllactosamine in Core 2 Branched O -Glycans : THE REQUIREMENT OF NOVEL β-1,4-galactosyltransferase IV AND β-1,3-N-acethylglucosaminyltransferase. Journal of Biological Chemistry. 273, 34843–34849 (1998). https://doi.org/10.1074/jbc.273.52.34843

23. Aoki, D., Appert, H.E., Johnson, D., Wong, S.S., Fukuda, M.N.: Analysis of the substrate binding sites of human galactosyltransferase by protein engineering. The EMBO journal. 9, 3171–8 (1990)

24. Miura, N., Ishida, N., Hoshino, M., Yamauchi, M., Hara, T., Ayusawa, D., Kawakita, M.: Human UDP-galactose translocator: molecular cloning of a complementary DNA that complements the genetic defect of a mutant cell line deficient in UDP-galactose translocator. J. Biochem. 120, 236–241 (1996). https://doi.org/10.1093/oxfordjournals.jbchem.a021404

25. Kabuss, R., Ashikov, A., Oelmann, S., Gerardy-Schahn, R., Bakker, H.: Endoplasmic reticulum retention of the large splice variant of the UDP-galactose transporter is caused by a dilysine motif. Glycobiology. 15, 905–911 (2005). https://doi.org/10.1093/glycob/cwi085

26. Wiertelak, W., Sosicka, P., Olczak, M., Maszczak-Seneczko, D.: Analysis of homologous and heterologous interactions between UDP-galactose transporter and beta-1,4-galactosyltransferase 1 using NanoBiT. Anal. Biochem. 593, 113599 (2020). https://doi.org/10.1016/j.ab.2020.113599

27. Bond, S.R., Naus, C.C.: RF-Cloning.org: an online tool for the design of restriction-free cloning projects. Nucleic Acids Research. 40, W209–W213 (2012). https://doi.org/10.1093/nar/gks396

28. Kudelka, M.R., Antonopoulos, A., Wang, Y., Duong, D.M., Song, X., Seyfried, N.T., Dell, A., Haslam, S.M., Cummings, R.D., Ju, T.: Cellular O-Glycome Reporter/Amplification to explore O-glycans of living cells. Nature Methods. (2015). https://doi.org/10.1038/nmeth.3675

29. Nakagawa, T., Takahashi, C., Matsuzaki, H., Takeyama, S., Sato, S., Sato, A., Kuroda, Y., Higashi, H.: N-glycan-dependent cell-surface expression of the P2Y2 receptor and N-glycan-independent distribution to lipid rafts. Biochemical and Biophysical Research Communications. 485, 427–431 (2017). https://doi.org/10.1016/J.BBRC.2017.02.061

30. Kuhn, P.-H., Voss, M., Haug-Kröper, M., Schröder, B., Schepers, U., Bräse, S., Haass, C., Lichtenthaler, S.F., Fluhrer, R.: Secretome Analysis Identifies Novel Signal Peptide Peptidase-Like 3 (SPPL3) Substrates and Reveals a Role of SPPL3 in Multiple Golgi Glycosylation Pathways. Molecular & Cellular Proteomics. 14, 1584–1598 (2015). https://doi.org/10.1074/mcp.M115.048298

31. Mikolajczyk, K., Kaczmarek, R., Czerwinski, M.: How glycosylation affects glycosylation: the role of N-glycans in glycosyltransferase activity. Glycobiology. cwaa041 (2020). https://doi.org/10.1093/glycob/cwaa041

32. Kellokumpu, S., Hassinen, A., Glumoff, T.: Glycosyltransferase complexes in eukaryotes: long-known, prevalent but still unrecognized. Cell. Mol. Life Sci. 73, 305–325 (2016). https://doi.org/10.1007/s00018-015-2066-0

33. Amann, T., Hansen, A.H., Kol, S., Lee, G.M., Andersen, M.R., Kildegaard, H.F.: CRISPR/Cas9-Multiplexed Editing of Chinese Hamster Ovary B4Gal-T1, 2, 3, and 4 Tailors *N* -Glycan Profiles of Therapeutics and Secreted Host Cell Proteins. Biotechnology Journal. 13, 1800111 (2018). https://doi.org/10.1002/biot.201800111

34. Ramakrishnan, B., Qasba, P.K.: Structure-based evolutionary relationship of glycosyltransferases: a case study of vertebrate β1,4-galactosyltransferase, invertebrate β1,4-N-acetylgalactosaminyltransferase and α-polypeptidyl-N-acetylgalactosaminyltransferase. Current Opinion in Structural Biology. 20, 536–542 (2010). https://doi.org/10.1016/j.sbi.2010.07.004

35. Harrus, D., Khoder-Agha, F., Peltoniemi, M., Hassinen, A., Ruddock, L., Kellokumpu, S., Glumoff, T.: The dimeric structure of wild-type human glycosyltransferase B4GalT1. PLoS ONE. 13, e0205571 (2018). https://doi.org/10.1371/journal.pone.0205571

36. Khoder-Agha, F., Sosicka, P., Escriva Conde, M., Hassinen, A., Glumoff, T., Olczak, M., Kellokumpu, S.: N-acetylglucosaminyltransferases and nucleotide sugar transporters form multi-enzyme–multi-transporter assemblies in golgi membranes in vivo. Cell. Mol. Life Sci. 76, 1821–1832 (2019). https://doi.org/10.1007/s00018-019-03032-5

37. Hassinen, A., Kellokumpu, S.: Organizational Interplay of Golgi N-Glycosyltransferases Involves Organelle Microenvironment-Dependent Transitions between Enzyme Homo- and Heteromers. J. Biol. Chem. 289, 26937–26948 (2014). https://doi.org/10.1074/jbc.M114.595058

38. Fukuta M., Habuchi O.: Keratan Sulfate Gal-6-Sulfotransferase. In: Taniguchi N. et al. (eds) Handbook of Glycosyltransferases and Related Genes. Springer, Tokyo (2002)

39. Haraguchi, M., Yamashiro, S., Furukawa, K., Takamiya, K., Shiku, H., Furukawa, K.: The effects of the site-directed removal of N-glycosylation sites from beta-1,4-N-acetylgalactosaminyltransferase on its function. Biochem J. 312, 273–280 (1995)

40. Kato, T., Suzuki, M., Murata, T., Park, E.Y.: The effects of N-glycosylation sites and the N-terminal region on the biological function of beta1,3-N-acetylglucosaminyltransferase 2 and its secretion. Biochem. Biophys. Res. Commun. 329, 699–705 (2005). https://doi.org/10.1016/j.bbrc.2005.02.033

41. Nagai, K., Ihara, Y., Wada, Y., Taniguchi, N.: N-glycosylation is requisite for the enzyme activity and Golgi retention of N-acetylglucosaminyltransferase III. Glycobiology. 7, 769–776 (1997). https://doi.org/10.1093/glycob/7.6.769

42. Baboval, T., Koul, O., Smith, F.I.: N-glycosylation site occupancy of rat alpha-1,3-fucosyltransferase IV and the effect of glycosylation on enzymatic activity. Biochim. Biophys. Acta. 1475, 383–389 (2000). https://doi.org/10.1016/s0304-4165(00)00094-5

43. Skropeta, D.: The effect of individual N-glycans on enzyme activity. Bioorganic & Medicinal Chemistry. 17, 2645–2653 (2009). https://doi.org/10.1016/j.bmc.2009.02.037

44. Lee, H.S., Qi, Y., Im, W.: Effects of N -glycosylation on protein conformation and dynamics: Protein Data Bank analysis and molecular dynamics simulation study. Sci Rep. 5, 1–7 (2015). https://doi.org/10.1038/srep08926

45. Uemura, S., Kurose, T., Suzuki, T., Yoshida, S., Ito, M., Saito, M., Horiuchi, M., Inagaki, F., Igarashi, Y., Inokuchi, J.I.: Substitution of the N-glycan function in glycosyltransferases by specific amino acids: ST3Gal-V as a model enzyme. Glycobiology. 16, 258–270 (2006). https://doi.org/10.1093/glycob/cwj060

46. Martina, J.A., Daniotti, J.L., Maccioni, H.J.F.: GM1 Synthase Depends on N-Glycosylation for Enzyme Activity and Trafficking to the Golgi Complex. 7

47. Malissard, M., Borsig, L., Marco, S., Grutter, M.G., Kragl, U., Wandrey, C., Berger, E.G.: Recombinant Soluble beta-1,4-Galactosyltransferases Expressed in Saccharomyces cerevisiae. Purification, Characterization and Comparison with Human Enzyme. Eur J Biochem. 239, 340–348 (1996). https://doi.org/10.1111/j.1432-1033.1996.0340u.x

**Table S1. List of utilized primers.**

| Name | Sequence 5ʹ - 3ʹ |
| --- | --- |
| B4GalT4_T6A | CATGGGCTTCAACCTGGCTTTCCACCTTTCCTAC |
| B4GalT4_T222A | GTTGGCAGGAACAGCGCTGGGTACAGGTTAC |
| B4GalT4_T337A | CAATCCTTTATATATCAACATCGCAGTGGATTTCTGGTTTG |
| RF-B4T4-F | ACCCTTATGATGTGCCAGACTATGCCATGGGCTTCAACCTGACTTT |
| RF-B4T4-R | TCTTATCATGTCTGGCCAGCTAGCTTCATGCACCAAACCAGAAATCC |
| gRNA_NH_5_sens: | CACCGcaacagtaatcggaatttgt |
| gRNA_NH_5_antisens: | AAACacaaattccgattactgttgC |
| gRNA_N_6_sens: | CACCGctttgtgcctgacagtggtt |
| gRNA_N_6_antisens: | AAACaaccactgtcaggcacaaagC |
| KOB4T4-1F | ATGGGCTTCAACCTGACTTTC |
| KOB4T4-1R | ACTCCTTTGCTTTAGGAATCTCTTG |
| KOB4T4-2F1 | GCAGAAAATCCCAAAGTGTCCA |
| KOB4T4-2F2 | CAGATCTCACTTTGGAAGAGGTACAG |
| KOB4T4-2R1 | TGCAGGAAGGGATGCAGAT |
| KOB4T4-2R2 | GCTGCCTCTGCAGGAAGG |
| LgBiT-SLC35A2_F | AAAACTCGAGAatggcagcggttgggg |
| LgBiT-SLC35A2_R | AAAAGAATTCTTActtcaccagcactgactttgg |
| SmBiT-B4GalT4_F | aaaaCTCGAGaATGGGCTTCAACCTGACTTTC |
| SmBiT-B4GalT4_R | aaaaGAATTCTCATGCACCAAACCAGAAATCC |

.

**TableS2. List of expression plasmids used in this study.**

| Name of construct | Backbone plasmid |
| --- | --- |
| pSelect_myc-6His-B4GalT4 | pSELECT-zeo - InvivoGen |
| pSelect_myc-6His-B4GalT4 S6A (M1) | pSELECT-zeo - InvivoGen |
| pSelect_myc-6His-B4GalT4 S222A (MT2) | pSELECT-zeo - InvivoGen |
| pSelect_myc-6His-B4GalT4 S331A (MT3) | pSELECT-zeo - InvivoGen |
| pSelect_myc-6His-B4GalT4 3xMut (MT123) | pSELECT-zeo - InvivoGen |
| pSelect_myc-6His-B4GalT4 T22A T331A (MT23) | pSELECT-zeo - InvivoGen |
| pSelect_NHA-B4GalT4 | pSELECT-NHA-zeo - InvivoGen |
| pSelect_NHA-MT2 | pSELECT-NHA-zeo - InvivoGen |
| pSelect_NHA-MT3 | pSELECT-NHA-zeo - InvivoGen |
| pSelect_NHA-MT23 | pSELECT-NHA-zeo - InvivoGen |
| px462 Puro _NH_5 | px462 - AddGene |
| px462 Zeo_N_6 | px462 - AddGene |
| sc-407251-NIC | N/A - Santa Cruz Biotechnology |
| pSelect_HA_blasti-B4T4 | pSELECT-CHis-blasti - InvivoGen |
| pSelect_HA_blasti-MT2 | pSELECT-CHis-blasti - InvivoGen |
| pSelect_HA_blasti-MT3 | pSELECT-CHis-blasti - InvivoGen |
| LgBiT-SLC35A2 | pBiT1.1-N[TK/LgBiT] - Promega |
| SmBiT-B4GalT4 | pBiT2.1-N[TK/SmBiT] - Promega |
| SmBiT-B4GalT4_MT2 | pBiT2.1-N[TK/SmBiT] - Promega |
| SmBiT-B4GalT4_MT3 | pBiT2.1-N[TK/SmBiT] - Promega |
| SmBiT-B4GalT4_MT23 | pBiT2.1-N[TK/SmBiT] - Promega |
| NEG | NanoBiT® Negative Control Vector |

**Table S3. List of antibodies used in this study.**

| Antibody | Clonality | Dilution IF / WB | Host | Company |
| --- | --- | --- | --- | --- |
| anti-c-myc | polyclonal | 1: 10 000 /1: 1000 | Chicken | Abcam |
| anti-Chicken-HRP | polyclonal | 1: 10 000 | Goat | Abcam |
| anti-calnexin | polyclonal | 1: 2000 / 1: 200 | Rabbit | Abcam |
| anti-Rabbit-HRP | polyclonal | 1: 10 000 | Goat | Sigma-Aldrich |
| anti-EEA1 | monoclonal | 1:1000 (WB) | Mouse | Santa Cruz |
| anti-HA-HRP | polyclonal | 1: 500 | Rabbit | Sigma-Aldrich |
| anti-GM130 | polyclonal | 1: 100 (IF) | Mouse | Abcam |
| anti-B4GalT4 | monoclonal | 1: 1000 (WB) | Rabbit | Abnova |
| anti-B4GalT4 | polyclonal | 1: 1000 (WB) | Rabbit | Proteintech |
| anti-keratan sulfate | monoclonal | 1:100 | Mouse | AMSBIO |
| anti-Chicken Alexa Fluor488 | polyclonal | 1: 200 (IF) | Goat | Thermo Fisher Scientific |
| anti-Rabbit Alexa Fluor568 | polyclonal | 1: 200 (IF) | Goat | Thermo Fisher Scientific |

*IF – immunofluorescence staining, WB – western blotting.

**Figure legends**

**Fig. 1. Glycosylation of overexpressed B4GalT4.** Western blotting analysis of lysates obtained from cells overexpressing variants of B4GalT4 treated (+) or not (-) with PNGase. (a) HEK293T were transfected with either non-mutated (myc-B4GalT4) or individual mutants (myc-MT1–3) and subsequently lysed and treated with PNGase F. Protein staining was performed with anti-*c*-myc antibody. Protein loading was assessed using staining with anti-calnexin antibody. (b) A375 B4GalT4 KO cells were transfected with non-mutated (HA-B4GalT4) or mutated (HA-MT1-3) versions of B4GalT4. As previously described, prior to SDS-PAGE analysis lysates were treated with PNGase F and protein bands were visualized with anti-HA antibody with loading control visualized using anti-calnexin antibody.

**Fig. 2. Subcellular localization of B4GalT4 versions in HEK293T cells.** Indirect staining of HEK293T cells stably overexpressing non-mutated (myc-B4GalT4) or mutated (myc-MT2-23) versions of B4GalT4 with anti-*c*-myc (green) and anti-GM130 (red) antibodies. Cell nuclei were counterstained with DAPI (blue). Scale bar –10 µm.

**Fig. 3. Secretion of different B4GalT4 versions.** HEK293T cells stably overexpressing variants of B4GalT4 were cultured in Opti-MEM without FBS for 24 h. Conditioned media were concentrated by centrifugation in Amicon Pro 10 kDa cut-off membranes and analyzed by Western blotting with anti-B4GalT4 and anti-Mgat5 antibodies for protein assessment and loading control, respectively.

**Fig. 4. Assessment of *in vitro* activity of B4GalT4.** HEK293T cells stably overexpressing variants of B4GalT4 were used as a source of protein in immunoprecipitation with magnetic beads. Purified proteins still immobilized on magnetic beads were used as a source of enzyme in reaction towards the indicated acceptor and resolved in HPLC conditions described in Materials and Methods. Commercially available recombinant B4GalT4 was used as a positive control to identify products of successful reactions.

**Fig. 5. A decrease in keratan sulfate synthesis in cell cultures depleted in endogenous B4GalT4.** Western blotting analysis of A375 cells depleted in endogenous B4GalT4 (B4GalT4 KO) and clonal cell cultures overproducing B4GalT4 versions.

**Fig. 6. Assessment of glycan impact on interaction between B4GalT4 and SLC35A2**. Results of the NanoBiT assay performed for the selected combinations and the corresponding negative control. B4GalT4-deficient HEK293T cell cultures were transfected with combinations of plasmids bearing either small or large subunit of the NanoLuc luciferase fused to studied proteins or a negative control (HaloTag fused with the small NanoLuc subunit). A2 – SLC35A2 tagged with the large NanoLuc subunit at the N-terminus;B4GalT4, M2, M3 and M23 – respective B4GalT4 variants tagged with the small NanoLuc subunit at the N-terminus, NEG -– HaloTag fused with the small NanoLuc subunit, RLU – relative luminescence unit. p < 0.0001 ****(b) Fold changes calculated by dividing the average luminescence obtained for the tested combinations (Sample RLU) by the average luminescence obtained for the corresponding negative control (Control RLU). The threshold value was set at 10, according to the manufacturer's suggestions.

**Fig. S1. Full subcellular localization of B4GalT4 versions in HEK293T cells.** Indirect staining of HEK293T cells stably overexpressing non-mutated (myc-B4GalT4) or mutated (myc-MT2-23) versions of B4GalT4 with anti-*c*-myc (green) and anti-organelle (red) antibodies. Cell nuclei were counterstained with DAPI (blue). Scale bar represents 10 µm.

**Fig. S2. Alignments of cDNA fragments of unchanged and knock-out cell cultures**. Mixtures of RT-PCR products obtained from HEK293T and A375 treated and unchanged cultures were cloned into the pJET1.2/blunt vector and sequenced.

**Fig. S3. MALDI-TOF analysis of O-glycans produced by the wild type and knock-out HEK293T and A375 cell cultures.** O-glycans from corresponding cell lines were isolated and labelled according to the procedure described in Materials and Methods.

**Fig. S4. MALDI-TOF analysis of desialylated N-glycans produced by the wild type and knock-out HEK29T and A375 cell cultures.** N-glycans from corresponding cell lines were isolated and labelled according to the procedure described in Materials and Methods.

**Fig. S5. Subcellular localization of B4GalT4 versions in A375 B4GalT4 KO cells.** Indirect staining of A375 B4GalT4 KO cells stably overexpressing non-mutated (myc-B4T4) or mutated (myc-MT2-23) versions of B4GalT4 with anti-*c*-myc (green) and anti-organelle (red) antibodies. Cell nuclei were counterstained with DAPI (blue). Intensity enhancement was done on all channels equally although intensity of composites was adjusted differently between images.

**Fig. S6 Effects of inhibition of proteasomal degradation on B4GalT4 versions**. (a) HEK293T cells stably expressing versions of B4GalT4 were treated for 6 h of culture with 8 µM MG132 prior to lysis and SDS-PAGE analysis with anti-c-myc and anti-calnexin antibodies for B4GalT4 visualization and assessment of protein loading. (b) SDS-PAGE analysis of cell lysates and solubilized proteins form insoluble pellets from HEK293T cells stably overexpressing versions of B4GalT4. (c, d) Similarly performed MG132 treatments for A375 cell line with B4GalT4 detection via anti-HA antibody and, in the case of non-precipitated samples, anti-EEA1 staining for loading control. (e) Indirect staining of HEK293T cells stably overexpressing different versions of B4GalT4 treated with MG132 as described above. Proteins were visualized with anti-*c*-myc antibody (green) and nuclei were counterstained with DAPI (blue). Scale bar represents 10 µm.
