# Supplementary material for: N-glycosylation of the human β1,4-galactosyltransferase 4 is crucial for its activity and Golgi localization
Source: Glycoconj J. 2020 Aug 22;37(5):577–88. doi: 10.1007/s10719-020-09941-z (PMC7501111; doi:10.1007/s10719-020-09941-z)

Supplemental Figure 1 - (Not to be Published)

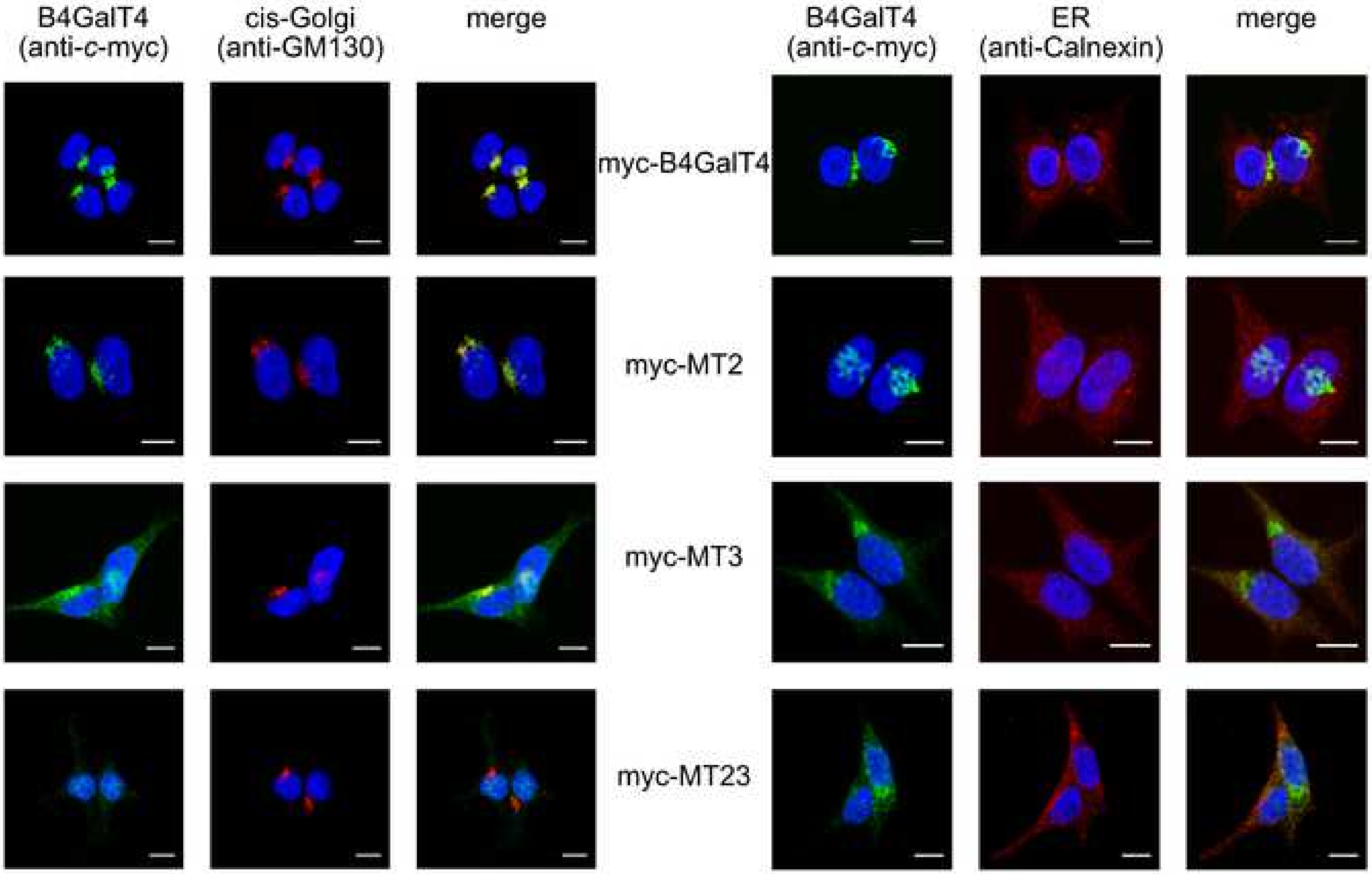

Supplemental Figure 2 (Not to be Published)

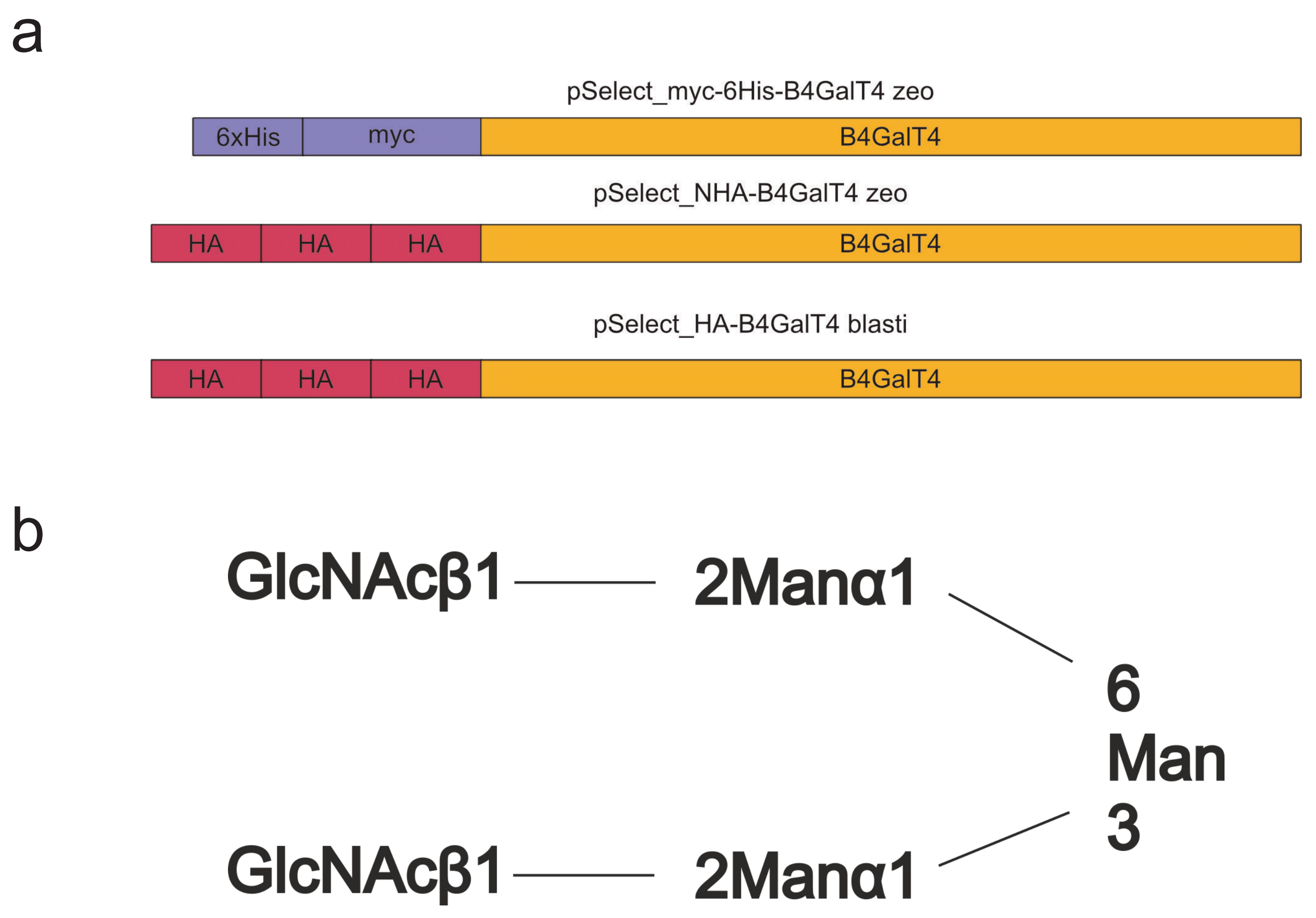

C

Alignment of *B4GalT4* CDS regions targeted for creation of gene A375 knock-out cell lines

```
B4GalT4(bp281-454)        CAGATCTCACTTTGGAAGAGGTACAGGCAGAAAATCCCAAAGTGTCCAGAGGCCGGTATC      60
A14.9-2_pJET1.2-for       CAGATCTCACTTTGGAAGAGGTACAGGCAGAAAATCCCAAAGTGTCCAGAGGCCGGTATC      60
A14.9-6_pJET1.2-for       CAGATCTCACTTTGGAAGAGGTACAGGCAGAAAATCCCAAAGTGTCCAGAGGCCGGTATC      60
A14.9-8_pJET1.-2-for      CAGATCTCACTTTGGAAGAGGTACAGGCAGAAAATCCCAAAGTGTCCAGAGGCCGGTATC      60

B4GalT4(bp281-454)        GCCCTCAGGAATGTAAAGCTTTACAGAGGGTCGCCATCCTCGTTCCCCACCGGAACAGAG      120
A14.9-2_pJET1.2-for       GCCCTCAGGAATGTAAAGCTTTACAGAGGGTCGCCATCCTCGTTCCCCACCGGAACAGAG      120
A14.9-6_pJET1.2-for       GCCCTGAGGAATGTAAAGCTTTACAGAGGGTCGCCATCCTCGTTCCCCACCGGAACAGAG      120
A14.9-8_pJET1.-2-for      GCCCTCAGGAATGTAAAGCTTTACAGAGGGTCGCCATCCTCGTTCCCCACCGGAACAGAG      120

B4GalT4(bp281-454)        AGAAACACCT--------------------------------------------------      130
A14.9-2_pJET1.2-for       AGAAACATCAGTCCCCACCGGAACAGAGAGA-----------------------------      151
A14.9-6_pJET1.2-for       AGAAACACCTGATGTACCGTCCCCACCGGAACAGAGAGAAACACCTGATGTACCCCCCAC      180
A14.9-8_pJET1.-2-for      AGAAACACCTGATGTACCCCCCACCGGAAC------------------------------      150

B4GalT4(bp281-454)        --------------------GATGTACCTGCTGGAACATCTGCATCCCTTCCTGCA  166
A14.9-2_pJET1.2-for       -------------AACACCTGATGTACCTGCTGGAACATCTGCATCCCTTCCTGCA  194
A14.9-6_pJET1.2-for       CGGAACAGAGAGAAACACCTGATGTACCTGCTGGAACATCTGCATCCCTTCCTGCA  236
A14.9-8_pJET1.-2-for      --------------------AGAGTACCTGCTGGAACATCTGCATCCCTTCCTGCA  186
```

Alignment of *B4GalT4* CDS regions targeted for creation of gene HEK293T knock-out cell lines

```
B4GalT4(bp1-140)           ATGGGCTTCAACCTGACTTTCCACCTTTCCTACAAATTCCGATTACTGTTGCTGTTGACT     60
H1.2.2-4_pJET1.2-for       ATGGGCTTCAACCTGACTTTCCACCTTTCC---------------------------TAC     33
H1.2.-2-5_pJET1.2-for      ATGGGCTTCAACCTGACTTTCCACCTTTCCTACAAATTCCGATTACTGTT----------     50
H1.2.2-6_pJET1.2-for       ATGGGCTTCAACCTGACTTTCCACCTTTCCTACAAATTC---------------------     39

B4GalT4(bp1-140)           TTGTGCCTGACAGTGGTTGGGTGGGCCACCAGTAACTACTTCGTGGGTGCCATTCAAGAG     120
H1.2.2-4_pJET1.2-for       ATTGCTGTTGACGTGGTTGGGTGGGCCACCAGTAACTACTTCGTGGGTGCCATTCAAGAG     93
H1.2.-2-5_pJET1.2-for      -----GCTGACAGTGGTTGGGTGGGCCACCAGTAACTACTTCGTGGGTGCCATTCAAGAG     105
H1.2.2-6_pJET1.2-for       -----CGATTAAGTGGTTGGGTGGGCCACCAGTAACTACTTCGTGGGTGCCATTCAAGAG     94

B4GalT4(bp1-140)           ATTCCTAAAGCAAAGGAGT       139
H1.2.2-4_pJET1.2-for       ATTCCTAAAGCAAAGGAGT       112
H1.2.-2-5_pJET1.2-for      ATTCCTAAAGCAAAGGAGT       124
H1.2.2-6_pJET1.2-for       ATTCCTAAAGCAAAGGAGT       113
```

Supplemental Figure 3 (Not to be Published)

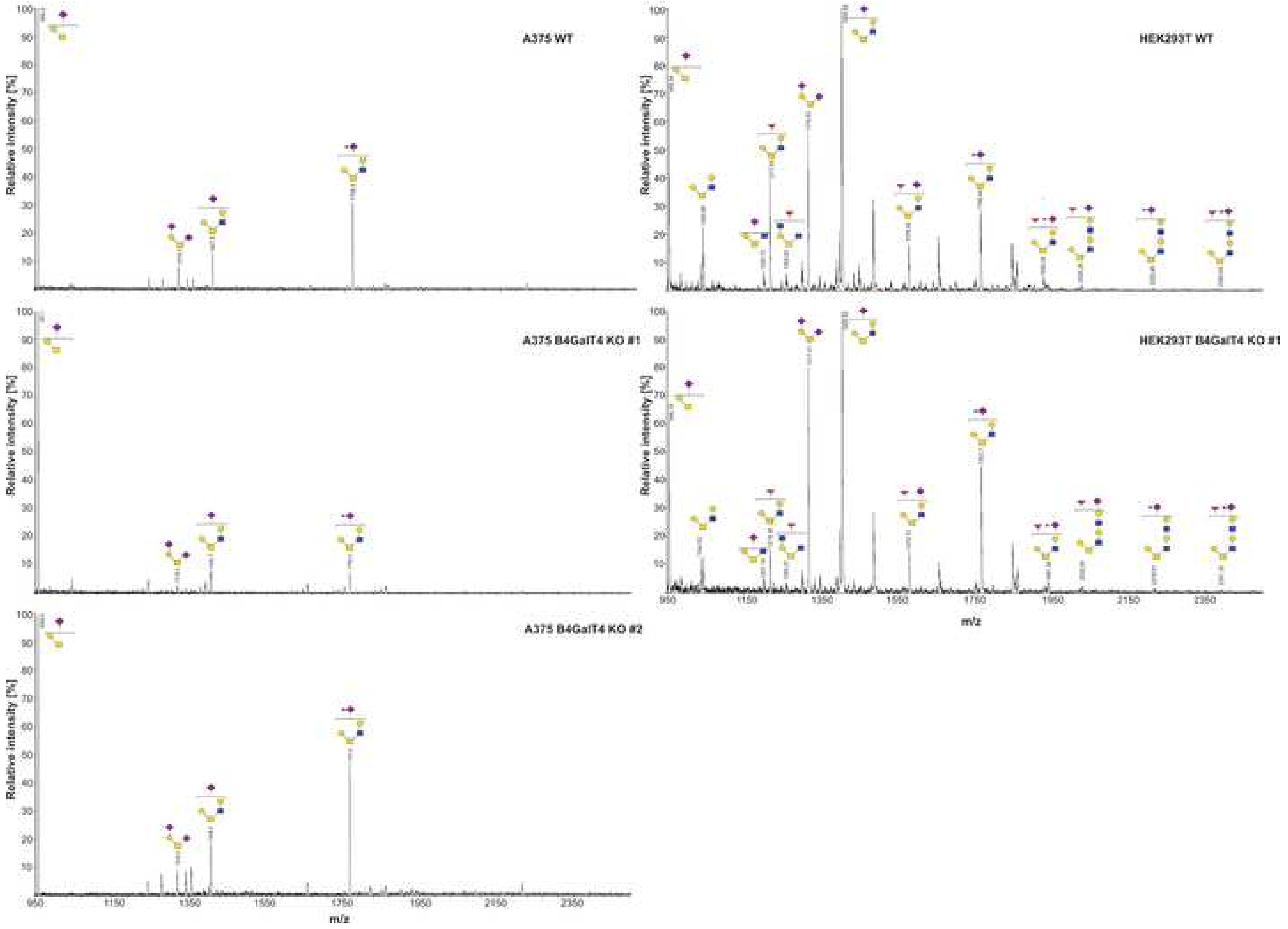

Supplemental Figure 4 (Not to be Published)

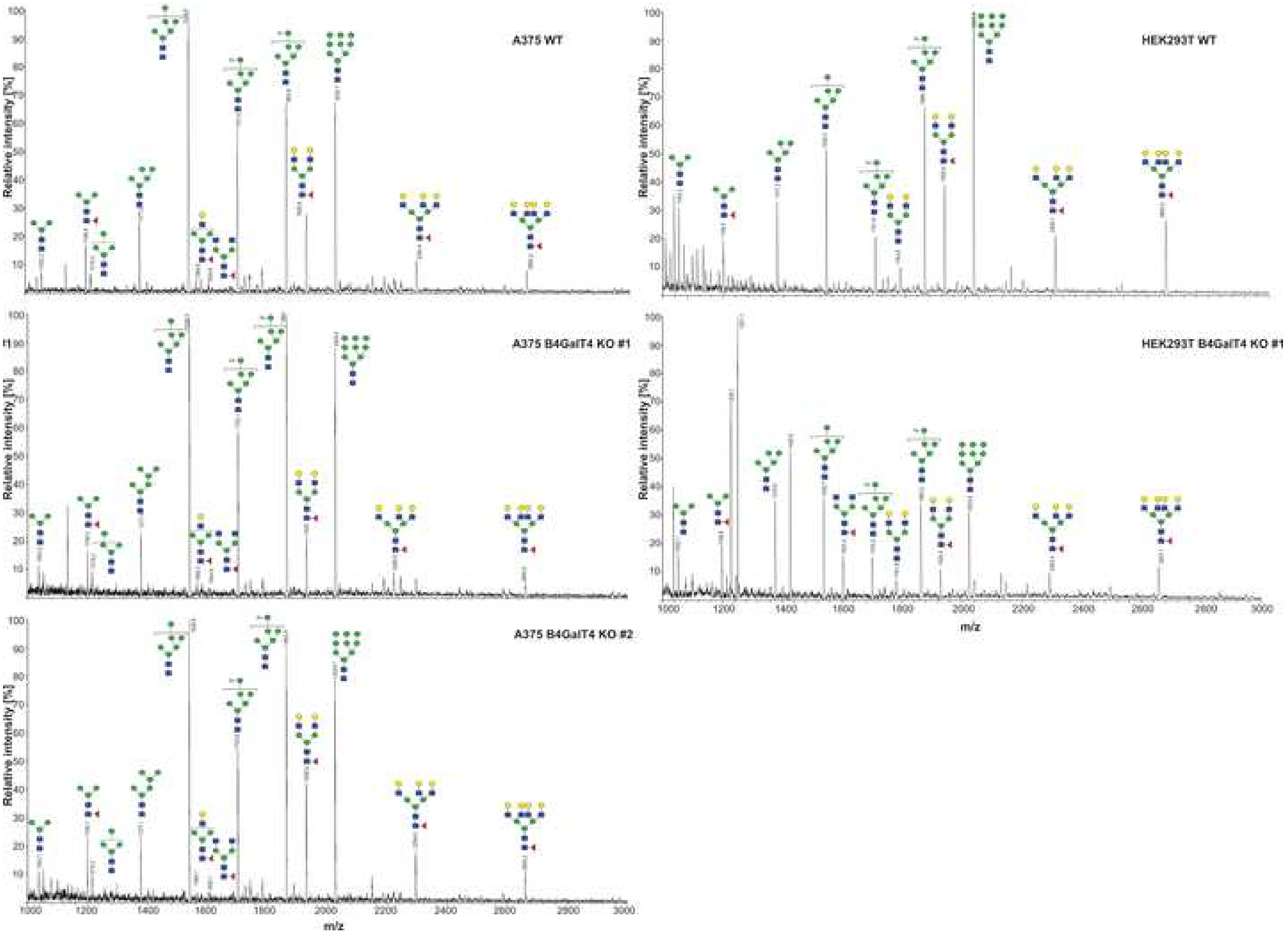

Supplemental Figure 5 (Not to be Published)

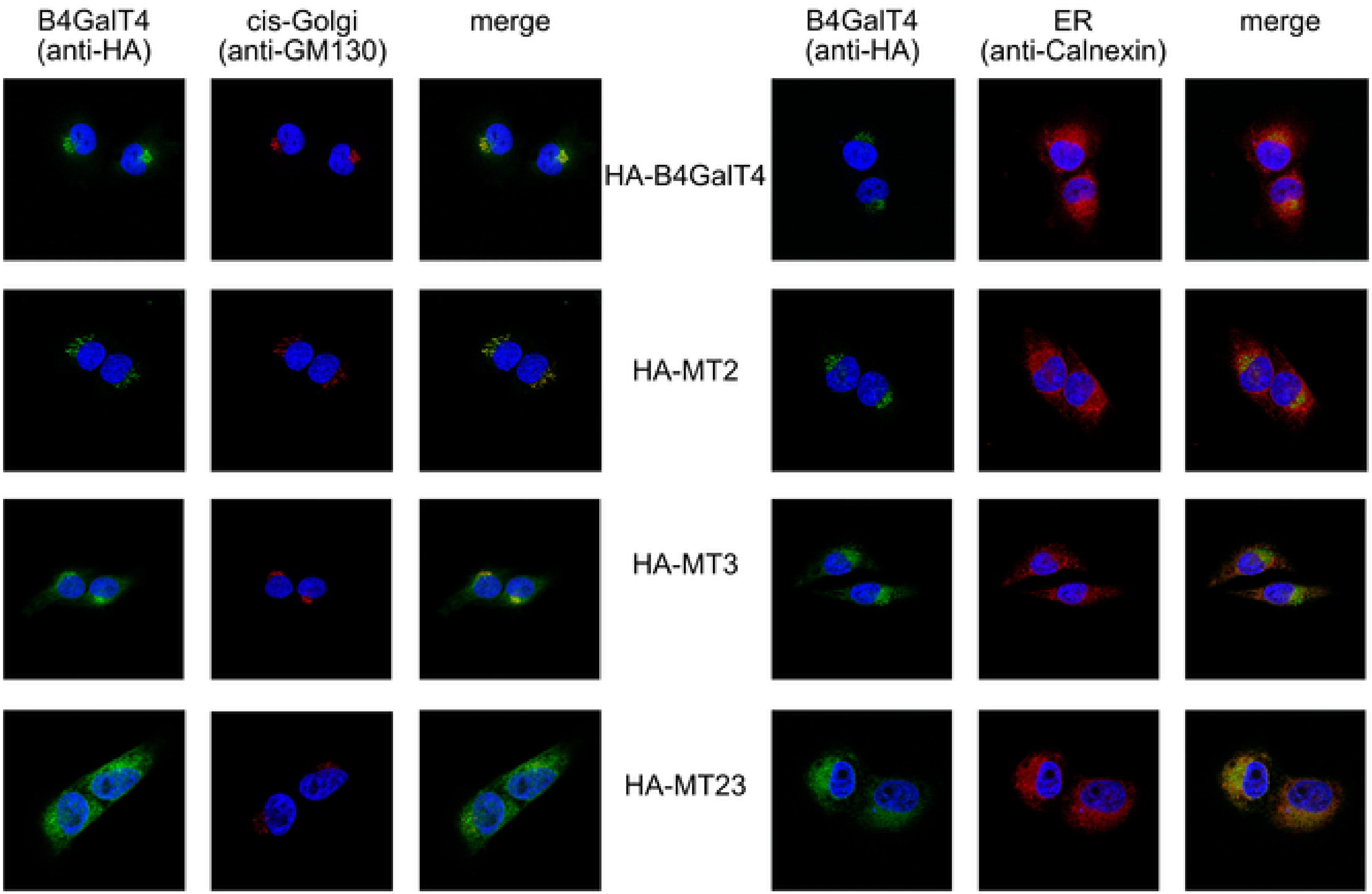

Supplemental Figure 6 (Not to be Published)

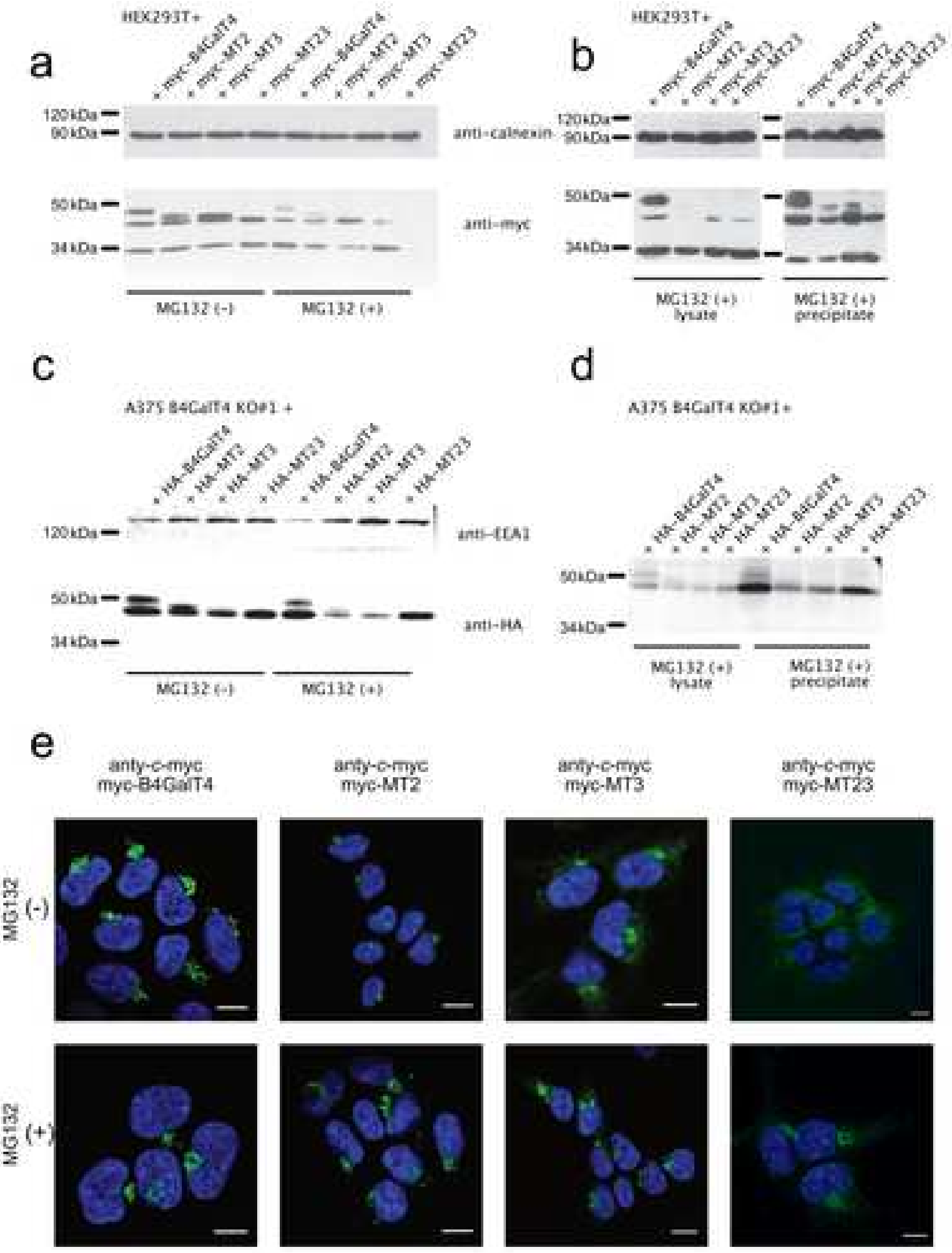

Supplement: Supplementary file 2 — (PDF 1785 kb) [file 10719_2020_9941_MOESM2_ESM.pdf]
